# Editorial: Timing the Brain: From Basic Sciences to Clinical Implications

**DOI:** 10.3389/fnhum.2022.880443

**Published:** 2022-03-22

**Authors:** Giuseppe Giglia, Dimitri Ognibene, Nadia Bolognini, Marina De Tommaso, Francesco Cappello, Pierangelo Sardo, Giuseppe Ferraro, Filippo Brighina

**Affiliations:** ^1^Department of Biomedicine, Neurosciences and Advanced Diagnostics, University of Palermo, Palermo, Italy; ^2^Department of Psychology, University of Milano-Bicocca, Milan, Italy; ^3^SMBNOS Department, University of Bari Aldo Moro, Bari, Italy

**Keywords:** timing, temporal binding window, time perception, Bayesian brain, cognition – multisensory integration – cortex

The concept of time has been a matter of debate in the field of physics, mathematics and philosophy for centuries (Renner and Stupar, 2017). While many physicists, such as Lee Smolin (2013), Nicolas Gisin (2020), point out the crucial role of time in quantum physics, already in 1922, Albert Einstein stated: “There is no such thing as the time of the philosopher” (Nathan, 2021). More recently, Di Biagio et al. (2021) argued that fundamental equations of elementary physics are time reversible and time can be ultimately considered as an illusion (Jaffe, 2018). While this will not inform that debate, it is evident that human's physiology presents a huge number of oscillating systems (i.e., circadian gene expressions, rithmic electrical signals in nervous system, pulsatile release of hormones) that divide the life cycle, synchronizing it but also keeping independent from environmental events, acting as internal clocks (Kondratova and Kondratov, 2012). Thus, from a biological perspective, time is a crucial reference frame for all perceptual, motor and cognitive processing in healthy subjects (Maccora et al., 2019), and in particular for the interaction with dynamic and social environments (Ognibene et al., 2013, 2019). Papers collected in the present topic highlight different aspects of the complex role of timing in brain function ranging from computational models to behavioral and neurophysiological evidence. This collection also offers an interesting perspective on how time is relevant and represents a common key for processing activity underlying neural functions taken as a whole, from elementary components (as sensory and motor functions) to more complex processes supporting cognition and behavior.

As argued by Pöppel et al. (1990), visual and auditory stimuli would hypothetically coincide only at about 10 m from a human observer, the so called “horizon of simultaneity”, however one of the most striking basic skills of the brain is the ability to merge signals coming from different sensory modalities into a single percept, despite the existing delays due to physical nature of the stimuli or different time courses of the involved nervous pathway and receptors transduction, that represents the real sense of multisensory perception.

Jagini reviewed literature focusing on the computational basis of the temporal binding in both multisensory and motor-sensory contexts under the framework of Bayesian causal inference models (Friston, 2010), underlying the role of precision of sensory likelihoods.

In this field, the work by Iida et al. showed that integration of visual-tactile stimuli contribute to body ownership in a time-dependent manner also for mirrored hand images, being much stronger in synchronous than in asynchronous condition. Yoshimatsu and Yotsumoto show a role of modality-specific top-down attention in the perception visual-auditory stimuli duration and presented a consistent hierarchical Bayesian model that computes multisensory duration through a weighted average of each sensory modality, where attention has a separate impact on the weight than signal reliability.

Similarly, the research report by Shukla and Bapi investigated the putative modulating role of numerical magnitude on time perception, according to the “Theory of Magnitude” (Walsh, 2003). Interestingly they did not find any modulation of temporal precision while temporal accuracy was affected by numerical magnitude thanks to attentional mechanisms.

Another cognitive function related to time perception, Temporal Information Processing (TIP), has been described as impaired in people affected by cognitive deficits, such as in aphasia (Szelag et al., 2014). On such a basis in their original research article, Choinski et al. investigated the role of TIP in shaping the relationship between language deficit and working memory. Thanks to an elegant study design, they showed that while verbal working memory is strongly affected by language deficit in both forward and backward tasks, TIP plays a crucial role in backward spatial working memory, considering its role as a logistic function (Jablonska et al., 2020). On the other hand, mental representation of the temporal aspects of an association is crucial also for associative learning; in this regard, the work by Chandran and Thorwart reviews evidence on temporal maps highlighting potential clinical implications.

Transcranial Alternating Current Stimulation (TACS) is a promising Non Invasive Brain Stimulation (NIBS) technique that uses time-alternating weak current in order to interact with the ongoing oscillatory activity in the stimulated cortex. While it has been found to be able to modulate motor performances in healthy subjects (Giustiniani et al., 2019), the research article by Giustiniani et al. failed to improve explosive power in sport subjects, adding also insights on genetic background.

Finally, timing plays a crucial role also in inducing neural plasticity as described by Guidali et al. Transcranial Magnetic Stimulation, another NIBS technique, can be effectively used to modulate neural plasticity in frontal and fronto-parietal networks; this can be achieved by targeting both peripheral-cortical and cortico-cortical routes in a time-dependent manner.

In conclusion, this collection highlights the role of time per se as a ubiquitous factor able to virtually modulate all brain processes. Many open questions remain to be unveiled by future research.

## Author Contributions

All authors listed have made a substantial, direct, and intellectual contribution to the work and approved it for publication.

## Conflict of Interest

The authors declare that the research was conducted in the absence of any commercial or financial relationships that could be construed as a potential conflict of interest.

## Publisher's Note

All claims expressed in this article are solely those of the authors and do not necessarily represent those of their affiliated organizations, or those of the publisher, the editors and the reviewers. Any product that may be evaluated in this article, or claim that may be made by its manufacturer, is not guaranteed or endorsed by the publisher.
